# RE-ENERGIZING GERONTOLOGY: BARRIERS TO PROGRAM GROWTH AND WHAT WE CAN DO ABOUT THEM

**DOI:** 10.1093/geroni/igae098.0815

**Published:** 2024-12-31

**Authors:** Judith Sugar, Harvey Sterns, Robert Maiden

**Affiliations:** Chair; Co-Chair; Discussant

## Abstract

With the growing population of older people in the U.S., it is more important than ever to increase the number of well-educated gerontology professionals. Beginning in 1972 with support from the Administration on Aging, resources were mobilized to establish and fund regional gerontology centers that would provide educational opportunities at all levels. Over the last 50 years, important work has been done to optimize the development of gerontology education. So it is ironic that at a time when we need more people to work with older adults, gerontology degree programs often struggle to enroll students. Low enrollments threaten the survival of existing programs. We have not done a good job marketing our field either inside or outside academia. We propose an effort to develop partnerships among like-minded organizations to promote the field and to attract funding to re-energize it. A coalition of partners needs to work with the employer community to convey the value of a gerontology education and ensure that graduates have the skills employers need. Accrediting gerontology programs and credentialing individuals can help to raise the profile of academic programs and graduates, and to assure employers that their employees bring the knowledge and skills to work with and for older clients, patients, and customers. The symposium presenters will engage attendees in a discussion of ways we can re-energize the field of gerontology to strengthen our academic programs and recruit more students to take advantage of the opportunities and meet the challenges of our aging population.

